# Salvianolic Acid B Improves Mitochondrial Function in 3T3-L1 Adipocytes Through a Pathway Involving PPARγ Coactivator-1α (PGC-1α)

**DOI:** 10.3389/fphar.2018.00671

**Published:** 2018-07-19

**Authors:** Yanyun Pan, Wenjing Zhao, Dandan Zhao, Chaoyang Wang, Na Yu, Tian An, Fangfang Mo, Jiaxian Liu, Jianan Miao, Bohan Lv, Yujie Gu, Sihua Gao, Guangjian Jiang

**Affiliations:** ^1^Diabetes Research Center, Beijing University of Chinese Medicine, Beijing, China; ^2^Traditional Chinese Medicine, Beijing University of Chinese Medicine, Beijing, China; ^3^Beijing Hospital of Traditional Chinese Medicine, Beijing, China; ^4^College of Acupuncture, Beijing University of Chinese Medicine, Beijing, China; ^5^Beijing Tian Tan Hospital, Capital Medical University, Beijing, China; ^6^Leonard Davis School of Gerontology, University of Southern California, Los Angeles, CA, United States

**Keywords:** salvianolic acid B, mitochondria respiration, glycolysis capacity, PPARγ, 3T3-L1 adipocytes, PGC-1α

## Abstract

**Purpose:** Mitochondrial dysfunction in adipose tissue has emerged as key to the development of obesity and diabetes. Salvianolic acid B (SalB) is a water-soluble ingredient derived from *Salvia miltiorrhiza* that has been shown to possess potential anti-obese and anti-diabetic activities. However, the cellular mechanism of SalB on mitochondrial function with respect to these metabolic disorders has not been elucidated. Therefore, we aim to investigate the effects of SalB on mitochondrial function in 3T3-L1 adipocytes and analyze the underlying molecular mechanism.

**Methods:** The effects of SalB on adipocyte differentiation, glucose uptake, and glycerol release were evaluated in 3T3-L1 adipocytes. Differentiated adipocytes were treated with SalB (50 μM) with or without PPARγ antagonist (GW9662, 20 μM) for 48 h, and mitochondrial oxygen consumption rate (OCR) as well as extracellular acidification rate (ECAR) were assessed using an XF Extracellular Flux Analyzer. The mitochondrial distribution of adipocytes was assessed using Mito Tracker Green (MTG) and observed under a fluorescent microscope. In addition, the mRNA expression levels of peroxisome proliferators-activated receptor γ/α (PPARγ/α), CCAAT/enhancer binding proteinα (C/EBPα), Nuclear respiratory factor 1/2 (NRF1/2), Uncoupling protein 2 (UCP2), and phosphofructokinase 2/fructose-2, 6-bisphosphatase 2 (PFKFB2) were detected by RT-PCR. Finally, changes in the protein levels of peroxisome proliferators-activated receptor γ coactivator-1α (PGC-1α) were determined by western blotting and immunofluorescence analysis.

**Results:** Treatment with SalB increased glucose uptake and mitochondrial respiration, reduced glycerol release and promoted adipocyte differentiation by increasing mRNA expression of adipogenic transcription factors including PPARγ, C/EBPα, and PPARα. Furthermore, SalB enhanced adipocytes mitochondrial content, mitochondrial respiration and glycolysis capacity, which had been attenuated by GW9662 treatment through the increased expression of PGC-1α.

**Conclusion:** Our results provide novel insights into the role of PGC-1α and mitochondria as probable mediators of SalB activity in the regulation of adipocyte differentiation in 3T3-L1 adipocytes.

## Introduction

Obesity is becoming a global public health problem due to the current lifestyle of energy-rich food consumption and physical inactivity, it is predicted that up to 58% of the world’s adult population will be overweight or obese by 2030 (Kelly et al., 2008; Tremmel et al., 2017). Obesity not only causes an array of chronic disorders, it also increases the risk of several metabolic diseases such as cardiovascular disease and diabetes, in which alterations in mitochondrial biogenesis, dynamics and function have been observed (Langin et al., 2005; Jelenik and Roden, 2013; Zamora-Mendoza et al., 2017). Therefore, an effective strategy to combat obesity may be to improve energy expenditure and metabolic efficiency of mitochondria in key metabolic organs, such as adipose tissue (Cedikova et al., 2016).

Mitochondria are essential organelles that provide energy for cellular metabolic activity in form of adenosine triphosphate (ATP) and are considered the “power house” of the cell (Zhang et al., 2007). In adipose tissue, obesity results in mitochondrial dysfunction with impaired oxidative phosphorylation and increased oxidative stress (Kim et al., 2008; Yin et al., 2014). Recently, multiple studies found that marked changes in mitochondrial mass, oxygen consumption, glycolysis capacity take place during adipogenesis programming and throughout the progression of metabolic disease (Zhang et al., 2013; Miller et al., 2015; Basse et al., 2017; Shiau et al., 2017). Since the adipocyte differentiation program requires large amount of ATP when cells become fully metabolically active, mitochondria are thought to be necessary regulators of this process (Heidari et al., 2018). Therefore, the modulation of mitochondrial oxygen consumption and glycolytic capacity may have therapeutic potential for the treatment of important pathophysiological conditions related to energy metabolism.

**FIGURE 1:**
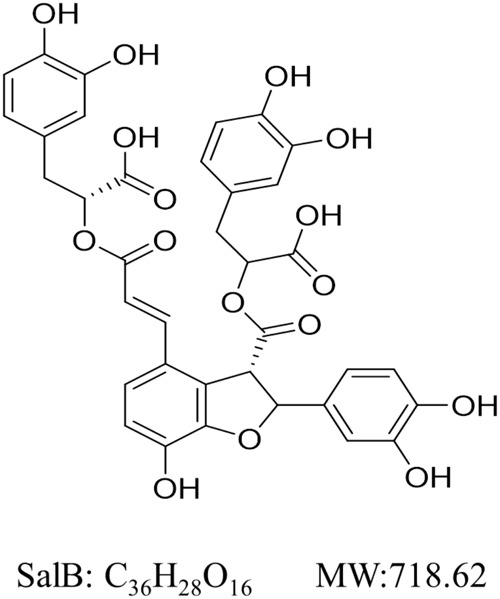
Chemical structure of Salvianolic acid B (SalB). Its molecular formula is C_36_H_28_O_16_ and molecular weight (MW) is 368.37 g/mol.

Salvianolic acid B (SalB, **Figure 1**) the most abundant and bioactive member of polyphenolic compounds derived from the root of *Salvia miltiorrhiza* (Danshen) and has been used widely and successfully in Traditional Chinese Medicine clinical practices for cardio-cerebral vascular diseases (Wang et al., 2013; Yu et al., 2016). Moreover, SalB is reported to have several therapeutic effects on obesity, insulin resistance and type 2 diabetes (Wang et al., 2014; Huang et al., 2016). Earlier studies have reported that SalB significantly decreases triglyceride and free fatty acid levels, increases serum SOD (a scavenger of free radicals) ability and decreases MDA (a by-product of lipid peroxidation) level, which reveal its potential anti-oxidative properties *in vivo* (Huang et al., 2015, 2016). Furthermore, it has been shown to moderate lipid metabolism in high-fat diet-induced obese mice by suppressing peroxisome proliferator activated receptor gamma (PPARγ)- mediated adipogenesis (Wang et al., 2014). SalB has also been found to improve the integrity of mitochondria and block mitochondria deformation and dysfunction induced by H_2_O_2_ (Xu et al., 2011; Liu et al., 2017). However, the effects of SalB on mitochondrial function in 3T3-L1 cells remain largely unknown. Here, we investigate the effects of SalB on mitochondrial function in 3T3-L1 adipocytes, and explore the potential regulatory mechanisms.

## Materials and Methods

### Reagents

SalB was purchased from Biopurify Phytochemicals Co., Ltd. (Cat. No. 15051803, Chengdu, China). Dulbecco’s modified Eagle’s medium (DMEM), Penicillin-streptomycin and 0.25%Trypsin-EDTA were purchased from Gibco (Cat. Nos. 1768707, 15140-122, and 1846496, Carlsbad, CA, United States). Fetal bovine serum (FBS) was purchased from Tianhang Biotechnology Co., Ltd. (Cat. No. HZ001, Hangzhou, China). Dexamethasone (DEX), insulin, and 3-isobutyl-1-methylxanthine (IBMX) were purchased from Sigma (Cat. No. I5500, d1756, and 17018, MO, United States). Oil Red O was purchased from Solarbio (Cat. No. G1260, Beijing, China). Glucose Oxidase Method kit was obtained from Applygen Technologies Inc. (Cat. No: E1010, Beijing, China). Enzychrom Glycerol Assay Kit was purchased from BioAssay Systems (Cat. No. EGLY-200, Hayward, CA, United States). GW9662 was bought from Selleck (Cat. No. S2915, Houston, TX, United States). Mito Tracker Green (MTG) was obtained from KeyGEN BioTECH (Cat. No. KGMP0072, Nanjing, China). The primers were designed and synthesized by Sangon Biotech (Shanghai, China). Antibody to PGC-1α was purchased from Proteintech (Cat. No. 20658-1-AP, Chicago, IL, United States). Fluorescein-Conjugated Goat anti-Rabbit lgG (H+L) was purchased from ZSGB-BIO (Cat. No. ZF0311, Beijing, China).

### Cell Culture and Differentiation

3T3-L1 preadipocytes were grown in DMEM supplemented with 10% FBS and 1% penicillin-streptomycin at 37°C in a humidified atmosphere with 5% CO2. After reaching 80% confluence, the cells were induced to differentiate in basic medium containing 10 μg/ml insulin, 0.5 mM IBMX and 0.4 μg/ml DEX for 2 days. The cell culture medium was subsequently exchanged with DMEM containing 10 μg/ml insulin for additional 2 days. Thereafter, the medium was replaced every 2 days until the cells exhibited an adipocyte phenotype. Various concentrations of SalB were added to the culture medium in differentiated adipocytes. The cell viability of SalB was determined using a Cell Counting Kit-8 (CCK-8) assay.

### Oil Red O Staining

In order to investigate the intracellular lipid accumulation, Oil Red O staining was performed. Briefly, differentiated 3T3-L1 adipocytes were treated with SalB (25, 50, and 100 μM). After 48 h, the cells were washed twice with PBS and fixed in 4% formaldehyde at room temperature for 30 min, then incubated for 1 h in filtered Oil Red O solution (isopropanol: water = 3:2). After staining, fat droplets were visualized as a “ring” and the images were obtained by light microscopy (Olympus, Japan). Oil Red O dye was eluted using isopropanol and the absorbance was determined by Microplate Reader.

### Detection of Glucose Concentrations and Glycerol Release in Differentiated Adipocytes

Differentiated 3T3-L1 adipocytes were incubated in DMEM without or with SalB (0, 25, 50, and 100 μM) for 48 h. Samples of the medium were collected and measured for glucose concentration and glycerol release using the Glucose Oxidase Method kit and Enzychrom Glycerol Assay Kit at 24 and 48 h according to the manufacturer’s instructions.

### Oxygen Consumption Rate (OCR) Measurement

3T3-L1 preadipocytes were seeded at 2 × 10^4^/well in XF^e^24 microplate coated with 0.2% gelation, the cells were differentiated as described above and treated with drugs for 48 h. The cells were washed twice and media was replaced with XF Assay medium containing 4.5 g/L glucose, 4.0 mM Glutamine and 1.0 mM Sodium pyruvate (adjust the pH to 7.35 ± 0.05 using 1 mol/L NaOH). The plates were placed in a 37°C incubator without CO_2_ for one hour prior to the assay. OCR measurements were performed using Seahorse Biosciences XF^e^ Analyzer. All experiments were performed at 37°C. After measurement of basal respiration, oligomycin (2.0 μM), FCCP (1.0 μM), rotenone/antimycin A (1.0 μM/1.0 μM) were added sequentially to measure ATP production, maximal respiratory, and non-mitochondrial respiration (NMR), respectively. These respiratory parameters of mitochondrial function were calculated as described previously (Mcgee et al., 2011).

### Extracellular Acidification Rates (ECARs) Measurement

Extracellular acidification rate was determined by monitoring glycolytic function and expressed in mpH/min. The measurement procedure was similar with OCR as described above. After measurement of basal ECAR, glucose solution (80 mM), oligomycin (5 mM), and 2-DG (100 mM) were added sequentially to determine glycolysis, glycolytic capacity, and glycolytic reserve. The parameters of glycolytic rates were analyzed as described previously (Mookerjee et al., 2016).

### Immunofluorescence Assay

Following exposure of 3T3-L1 cells to SalB (50 μM) and/or GW9662 (20 μM) for 48 h, the cells were washed with PBS, fixed in 4% paraformaldehyde, and permeabilized with 0.2% Triton-X 100 at room temperature. They were then blocked with 10% normal goat serum for 1 h and incubated overnight at 4°C with PGC-1α antibody (1:100 dilution). The secondary antibody, goat anti-rabbit FITC-conjugated (1:50 dilution) was added to the cells followed by incubation for 60 min. Nuclei were stained with DAPI for 5 min and viewed under confocal laser scanning microscope (Zeiss LSM 780). The fluorescence intensity of PGC-1α was calculated using Image Pro Plus software.

### Western Blotting Analysis

3T3-L1 adipocytes were lysed in RIPA buffer with protease inhibitor cocktail and quantified using the BCA Protein Assay Kit. Twenty micrograms of total protein per well was separated by SDS-PAGE. The protein was then transferred to PVDF membranes, and then incubated overnight at 4°C with antibodies of PGC-1α (1:500 dilution). Secondary goat anti-rabbit immunoglobulin G antibody was incubated at a dilution of 1:1000 for 1 h. Protein bands were visualized using ImageJ software. β-actin (1:1000 dilution) was used as the internal control.

### Mitochondrial Staining

3T3-L1 adipocytes (after 8 days of differentiation, in glass bottom cell culture dish) were treated with SalB (50 μM) and/or GW9662 (20 μM) for 48 h. The cells were then incubated with 200 nM MTG in low-glucose DMEM medium for 30 min at incubator. Mitochondria were viewed under confocal laser scanning microscope. The intensity of MTG fluorescence was analyzed using Image Pro Plus 6.0 software.

### RNA Isolation and Real-Time PCR

Total RNA was isolated from 3T3-L1 adipocytes using TriPure reagent (Roche, Indianapolis, IN, United States) according to the manufacturer’s protocols. Following isolation, 2 μg total isolated RNA was reverse-transcribed to synthesized cDNA using commercial kit. Subsequently, the diluted cDNA was used as the template to amplify the target genes. The samples were analyzed on an ABI 7500 Sequence Detection System. The primers sequences used in the experiments were summarized in **Table 1**. All PCR results were normalized to ARBP gene expression.

**Table 1:** Sequences of RT-PCR primers.

Gene	Forward (5′–3′)	Reverse (5′–3′)
C/EBα	CCAGAGGATGGTTTCGGGTC	TCCCCAACACCTAAGTCCCT
PPARα	GCGTACGGCAATGGCTTTAT	GAACGGCTTCCTCAGGTTCTT
PPARγ	CCATTCTGGCCCACCAACTT	CCTTCTCGGCCTGTCGATCC
PGC-1α	CCCTGCCATTGTTAAGACC	TGCTGCTGTTCCTGCTCCT
NRF-1	AGCACGGAGTGACCCAAAC	TGTACGTGGCTACATGGACCT
NRF-2	GGACATGGAGCAAGTTTGGC	GGGCTGGGGACAGTGGTAGT
UCP2	ACTGTGCCCTTACCATGCTCC	ATTGGTAGGCAGCCATTAGGG
PFKFB2	CTAACACGCTACCTCAACTGG	GACTATCTTGCTCTGGATGTAG
ARBP	TTTGGGCATCACCACGAAAA	GGACACCCTCCAGAATTTTC

### Statistical Analysis

All data were plotted as mean ± SEM using Graph Pad Prism Software (Version 6.0). One-way ANOVA test was performed between multiple group comparisons, followed by Dunnett’s test for the comparison between two groups. Differences were considered statistically significant at *P* < 0.05.

## Results

### Effect of SalB on Cell Viability and Adipogenic Differentiation in 3T3-L1 Cells

**FIGURE 2:**
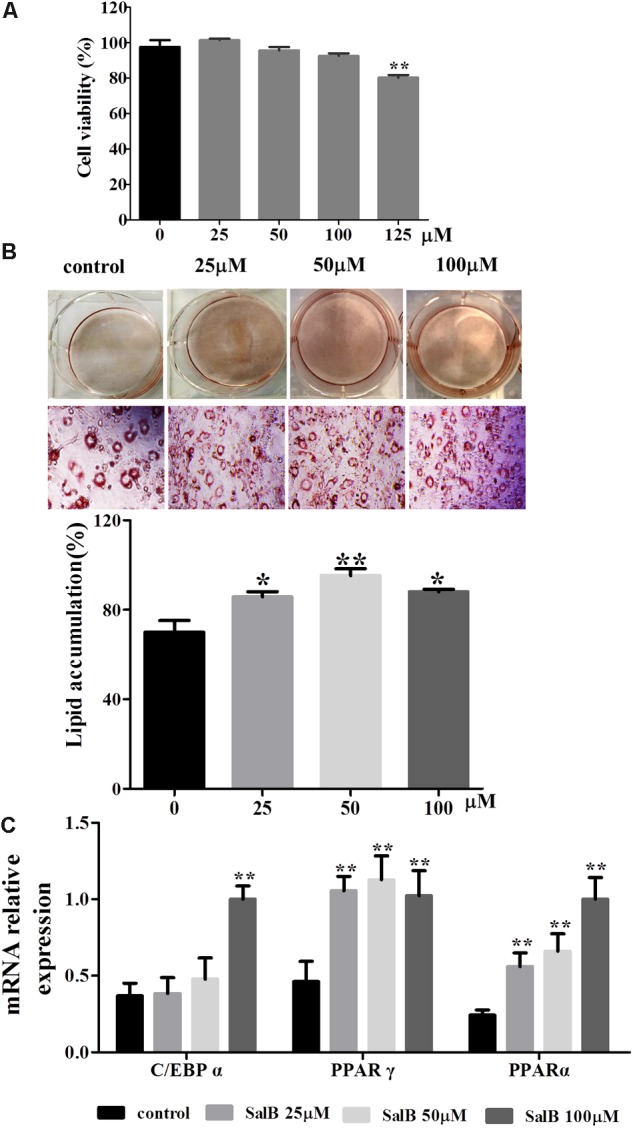
Effects of SalB on cell viability and adipogenic differentiation in 3T3-L1 preadipocytes. **(A)** Cell viability was determined with CCK-8 reagent after SalB treatment for 48 h. **(B)** Oil Red O staining of various concentrations of SalB-treated 3T3-L1 adipocytes (magnification: 20×). **(C)** The mRNA relative expression of C/EBPα, PPARγ, and PPARα after SalB treatment in 3T3-L1 adipocytes. Data are presented as mean ± SEM. ^∗^*p* < 0.05 and ^∗∗^*p* < 0.01 versus control group.

The cell viability assay was simply and accurately determined using the CCK-8 reagent. As shown in **Figure 2A**, there was no significant difference in adipocyte viability at concentrations of 25, 50, and 100 μM of SalB compared with the control group (*P* > 0.05). In contrast, cell viability decreased by 17.8% when the cells were incubated in medium with 125 μM SalB (*P* < 0.01). 3T3-L1 preadipocytes were incubated with the cocktail method without or with SalB (0, 25, 50 and 100 μM) for 8 days, and the accumulated oil droplets were visualized by staining with Oil Red O to identify differentiated cells. Based on microscopic observation, treatment with different concentrations of SalB promoted adipocyte differentiation and contributed to the accumulation of intracellular lipid droplets (**Figure 2B**). In this study, various doses of SalB also increased the mRNA expression of adipogenic transcription factors (**Figure 2C**). In detail, the expression of C/EBPα increased by 170.8% with the addition of 100 μM SalB (*P* < 0.01), while 25 and 50 μM SalB increased C/EBPα expression of 41.0 and 29.4%, respectively, compared to control group (*P* > 0.05). Furthermore, the expressions of PPARγ increased by 128.5, 143.9, and 121.4% (*P* < 0.01) and the expressions of PPARα increased by 80.8, 112.8, and 184.5% (*P* < 0.01), at dose of 25, 50, and 100 μM, respectively (**Figure 2D**).

### Effects of SalB on Glucose Uptake and Glycerol Release in Differentiated 3T3-L1 Adipocytes

**FIGURE 3:**
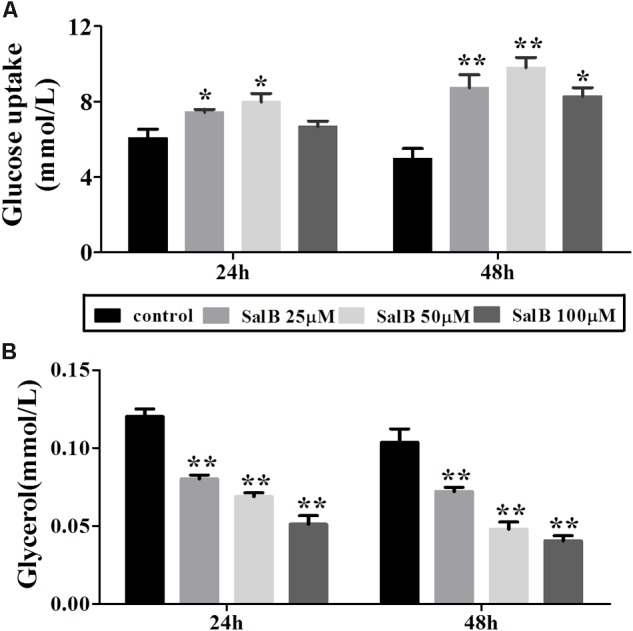
Effects of SalB on glucose uptake **(A)** and glycerol release **(B)** when the cells were incubated in medium with 25, 50, and 100 μM SalB for 24 and 48 h. Data are represented as mean ± SEM. ^∗^*p* < 0.05 and ^∗∗^*p* < 0.01 versus control group.

Glucose uptake increased by 22.7 and 28%, respectively, when the cells were incubated in medium with 25 and 50 μM SalB for 24 h. However, the higher dose SalB (100 μM) did not significantly increase (10.4%) glucose uptake compared to the control group (*P* > 0.05). After 48 h treatment, the glucose uptake increased by 75.8, 97.4, and 66.6% at dose of 25, 50, and 100 μM (**Figure 3A**). Of note, the optimal dose of SalB that increased glucose uptake was at 50 μM when the cells were incubated in medium for 24 h and 48 h. In addition, SalB treatment significantly reduced glycerol release by 33.2, 42.8, and 57.4% when the cells were incubated in medium with 25, 50, and 100 μM SalB for 24 h. Moreover, glycerol release was reduced by 30.4, 53.8, and 60.8% when the cells were incubated in medium with 25, 50, and 100 μM SalB for 48 h (**Figure 3B**). These results suggest that SalB could regulate obesity induced glycolipid metabolic disorders.

### Effects of SalB on Mitochondrial Respiratory Function in 3T3-L1 Adipocytes

**FIGURE 4:**
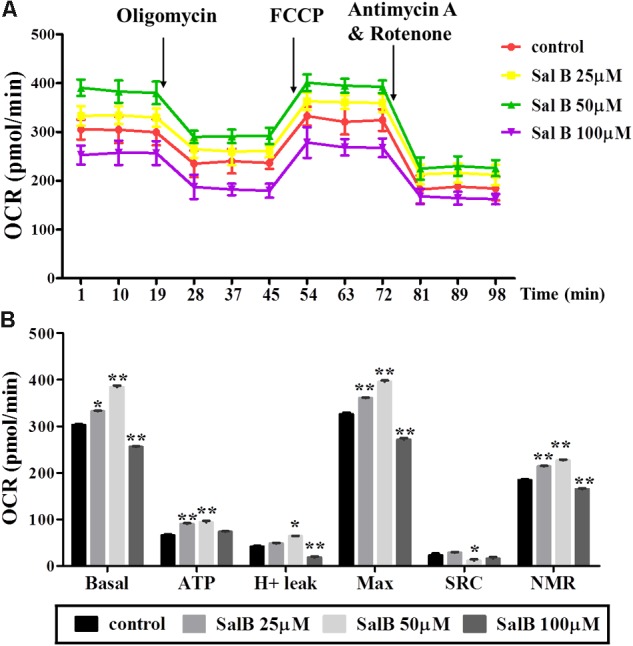
Effects of SalB (25, 50, and 100 μM) on oxygen consumption rate (OCR) in 3T3-L1 adipocytes. **(A)** Represents mitochondrial OCR curves obtained from different conditions. **(B)** Represents: Basal, basal respiration; ATP, ATP production; Max, maximum respiration; SRC, spare respiration capacity; H^+^ leak; and NMR, non-mitochondrial respiration of 3T3-L1 adipocytes under different treatments, respectively. Data are represented as mean ± SEM. ^∗^*p* < 0.05 and ^∗∗^*p* < 0.01 versus control group.

To explore the effect of SalB on mitochondrial respiration, OCR was measured using a Seahorse XF analyzer in 3T3-L1 adipocytes. The OCR of cell treated with SalB (25 and 50 μM) treatment remained higher than control group, but high dose SalB (100 μM) decreased the OCR in 3T3-L1 cells (**Figure 4A** and **Supplementary Table S1**). In detail, as shown in **Figure 4B**, basal mitochondrial respiration of SalB treated adipocytes were increased by 9.68% (*P* < 0.05) and 26.9% (*P* < 0.01), respectively, when the cells were incubated in medium with 25 and 50 μM SalB, while 100 μM SalB treated cells reduced it by 15.6% (*P* < 0.01) compared to control group. ATP production of SalB concentration 25, 50, and 100 μM increased by 37.5% (*P* < 0.01), 42.7% (*P* < 0.01), and 11.2% (*P* > 0.05), respectively. The H^+^ leak of SalB treated cells at concentrations of 25 and 50 μM was increased by 16.1% (*P* > 0.05) and 54.4% (*P* < 0.01), respectively, and 100 μM SalB reduced it by 56.4% (*P* < 0.01). The maximal mitochondrial respiration of SalB treated 3T3-L1 adipocytes was increased by 10.8% (*P* < 0.01) and 22.4% (*P* < 0.01), respectively, at dose of 25 and 50 μM, while 100 μM SalB decreased it by 16.7% (*P* < 0.01). The spare respiration capacity at a SalB concentration of 25 μM was increased by 24.6% (*P* > 0.05), but treatment with 50 and 100 μM SalB reduced it by 59.6% (*P* < 0.01) and 44.8% (*P* > 0.05), respectively. Treatment with 25 and 50 μM SalB increased NMR by 15.7% (*P* < 0.01) and 22.9%(*P* < 0.01), respectively. However, 100 μM SalB reduced it by 10.7% (*P* < 0.01).

### Effects of SalB on Mitochondria Respiration After Inhibiting PPARγ Activity

**FIGURE 5:**
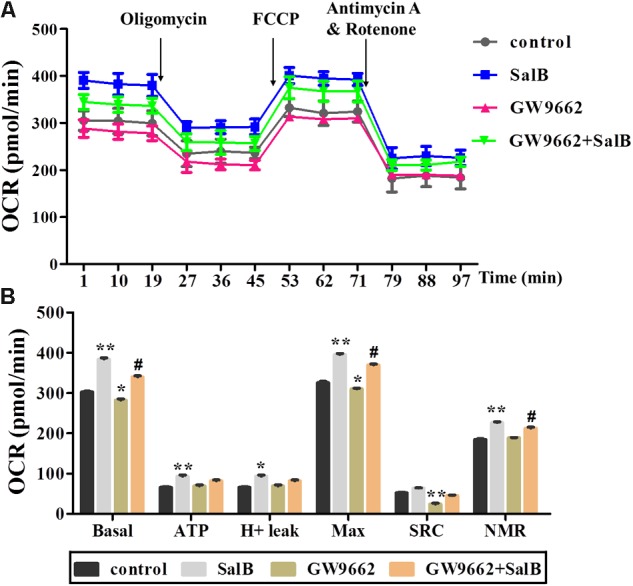
Effects of SalB (50 μM) and/or GW9662 (20 μM) on oxygen consumption rate (OCR) in 3T3-L1 cells. **(A)** Represents OCR curves obtained from different conditions. **(B)** Represents: Basal, basal respiration; ATP, ATP production; Max, maximum respiration; SRC, spare respiration capacity; H+ leak; and NMR, non-mitochondrial respiration of 3T3-L1 adipocytes under different treatments, respectively. Data are represented as mean ± SEM of three independent experiments. ^∗^*p* < 0.05 and ^∗∗^*p* < 0.01 versus control group, ^#^*p* < 0.01 and ^##^*p* < 0.01 versus GW9662 group.

To further explore the effects of SalB on mitochondrial function through the regulation of PPARγ activity in 3T3-L1 adipocytes. PPARγ antagonist (GW9692) was added with or without SalB. As shown in **Figure 5**, treatment with GW9692 inhibited the mitochondrial respiration function of differentiated 3T3-L1 adipocytes as expected (**Supplementary Table S2**). The positive effect of SalB on mitochondrial respiration was attenuated by treatment with the PPARγ antagonist.

### Effects of SalB on Glycolytic Function in 3T3-L1 Adipocytes After Inhibiting PPARγ Activity

**FIGURE 6:**
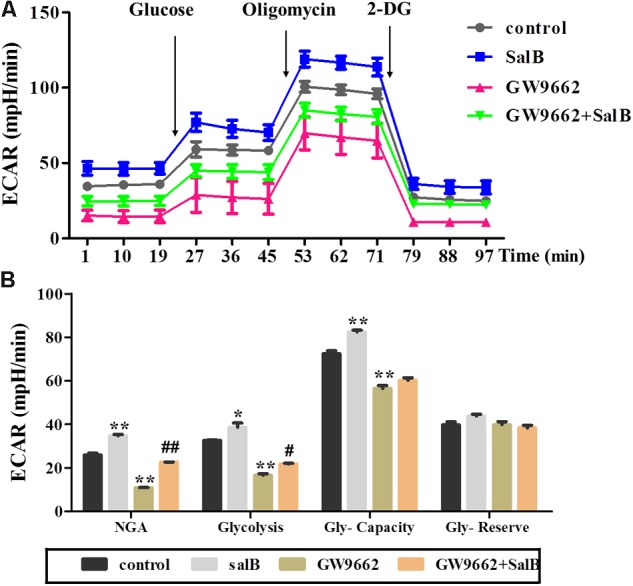
Effects of SalB (50 μM) and/or GW9662 (20 μM) on extracellular acidification rate (ECAR) in 3T3-L1 cells. **(A)** Represents ECAR curves obtained from different conditions. **(B)** Represents: NGA, non-glycolytic acidification; Gly-Capacity, glycolysis, glycolytic Capacity; and Gly-reserve, glycolytic reserve of 3T3-L1 adipocytes under different treatments, respectively. Data are represented as mean ± SEM. ^∗^*P* < 0.05 and ^∗∗^*P* < 0.01 versus control group, ^#^*P* < 0.05 and ^##^*P* < 0.01 versus GW9662 group.

**FIGURE 7:**
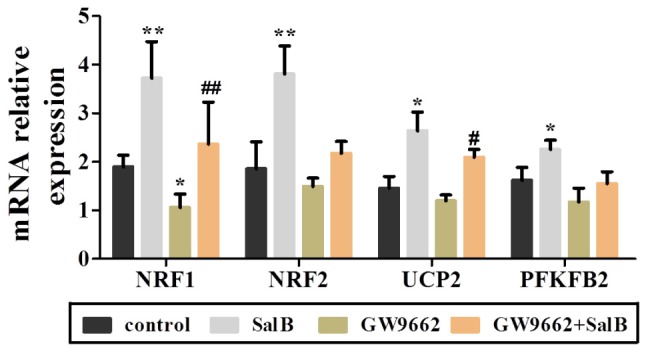
Effects of SalB (50 μM) and/or GW9662 (20 μM) on mitochondria related genes expression. Data are represented as mean ± SEM. ^∗^*P* < 0.05 and ^∗∗^*P* < 0.01 versus control group, ^#^*P* < 0.05 and ^##^*P* < 0.01 versus GW9662 group.

Extracellular acidification rate was used to determine the changes in the glycolytic rate in 3T3-L1 adipocytes (**Figure 6A** and **Supplementary Table S3**). Treatment with 50 μM SalB for 48 h markedly enhanced the basal rates of glycolysis and glycolytic capacity by 18.3 and 12.1%, respectively (*P* < 0.05). In addition, treatment with 20 μM GW9692 resulted in a significant reduction in glycolysis and glycolytic capacity by 97.2% (*p* < 0.01) and 28.3% (*p* < 0.01), respectively, following oligomycin injection. Interestingly, these two parameters were increased after supplementation with SalB compare to the GW9662 group (**Figure 6B**). However, the glycolytic reserve of ECAR exhibited no significant differences (*P* > 0.05). We further investigated the genes related to mitochondrial OxPhos and glycolysis. As shown in **Figure 7**, we found that SalB increased mRNA expression of NRF1, NRF2, UCP2, and PFKFB2 by 96.0, 104.8, 80.9, and 39.2%, respectively, compared with the control group. GW9662 treatment decreased the expression of these genes by 44.1, 19.5, 17.9, and 27.6%, respectively. Furthermore, GW9662 combined with SalB increased the expression of these genes by 122.7, 45.7, 75.1, and 32.5%, respectively, compared with the GW9662 group. These results suggest that SalB can improve mitochondrial glycolytic function through upregulating the related genes expression after inhibiting the PPARγ activation.

### Effects of SalB on PGC-1α Expression in 3T3-L1 Adipocytes

**FIGURE 8:**
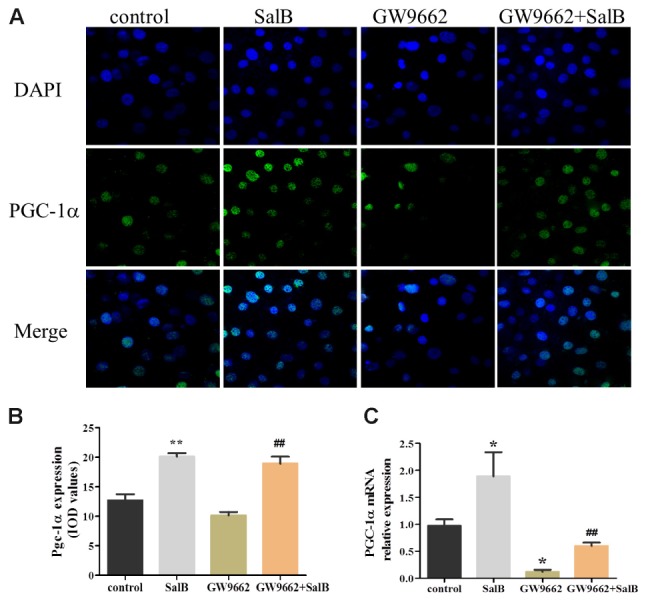
Effects of SalB (50 μM) and/or GW9662 (20 μM) on PGC-1α nuclear translocation **(A,B)** and protein expression level **(C)** and in 3T3-L1 adipocytes. The green color represents PGC-1α staining, the blue color represents nuclei staining, and the cyan (greenish–blue) color represents nuclear translocation. Data are represented as mean ± SEM. ^∗^*P* < 0.05 and ^∗∗^*P* < 0.01 versus control group, ^##^*P* < 0.01 versus GW9662 group.

As shown in **Figure 8**, PGC-1α was retained in the nucleus of 3T3-L1 cells as demonstrated by immunofluorescent. And SalB treatment increased the intensity and extent of staining for PGC-1α. Compared with the control group, SalB treatment increased the fluorescent density by 58.3% (*P* < 0.01) and GW9662 treatment reduced it by 20.0% (*P* > 0.05). GW9662 combined with SalB significantly increased fluorescence density by 86.1% (*P* < 0.01) compared with GW9662 group. In addition, SalB treatment significantly increased the protein expression of PGC-1α by 79.0% (*P* < 0.01), however, GW9662 treatment reduced its expression level by 35.0% (*P* < 0.05) compared with the control group. Furthermore, PGC-1α expression was increased approximately 1.63-fold after supplemented with SalB compared to GW9662 group (*P* < 0.01). These results indicate that SalB increase the expression of PGC-1α and ameliorate damage induced by PPARγ antagonist.

### Effects of SalB on Mitochondria Staining in 3T3-L1 Adipocytes

**FIGURE 9:**
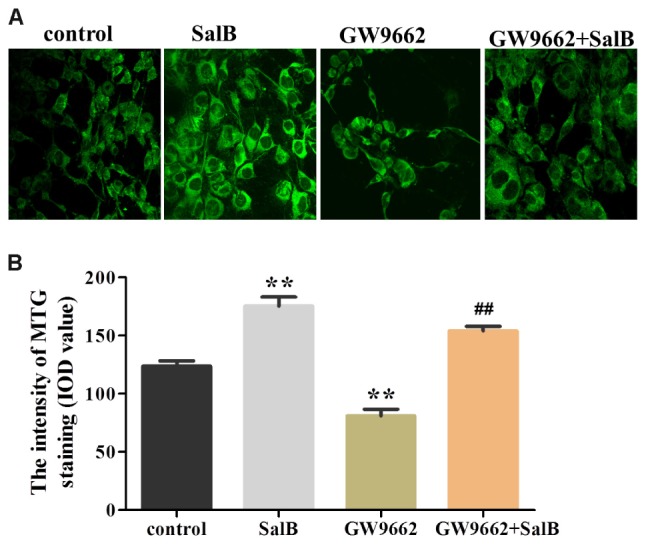
Effects of SalB (50 μM) and/or GW9662 (20 μM) on **(A)** adipocytes mitochondria and **(B)** in 3T3-L1 adipocytes. The green color represents mitochondria staining. Mitochondrial Data are represented as mean ± SEM. ^∗^*P* < 0.05 and ^∗∗^*P* < 0.01 versus control group, ^##^*P* < 0.01 versus GW9662 group.

To further investigate the effects on SalB on mitochondrial distribution, we stained adipocytes mitochondria using MTG reagent. As shown in **Figure 9**, treatment with SalB in 3T3-L1 cells increased the fluorescence intensity by 41.9% (*P* < 0.01), however, GW9662 significantly reduced it 34.6% (*P* < 0.01) compared with the control group. When GW9662 combined with SalB, the adipocyte mitochondrial fluorescence intensity increased by 90.4% compared to GW9662 group (*P* < 0.01).

## Discussion

Recently, more research has revealed that many diseases such as obesity, diabetes, neurodegenerative diseases, aging, and cardiovascular diseases have a close relationship with mitochondrial function (Heilbronn et al., 2007; Sivitz and Yorek, 2010; Jelenik and Roden, 2013). Mitochondria are the main site for glycolipid metabolism and oxygen consumption, which are associated with the basal metabolic rate (Cruz et al., 2018). Therefore, improving mitochondrial function of adipocytes may be strategy to prevent obesity and obesity-related metabolic complications. SalB has been shown to exert protective effects on obesity and diabetes (Semple et al., 2004; Huang et al., 2016), although its potential mechanism on mitochondrial function is not clear. In this study, we demonstrated that SalB treatment increased glucose uptake, reduced glycerol release, enhanced adipocytes glycolysis capacity, mitochondrial respiration, and increased the expression of related genes including NRF1, NRF2, UCP2, and PFKFB2.

Here, we aimed to investigate whether SalB treatment combined with PPARγ antagonists could further alter mitochondrial morphology, content, respiratory, and glycolytic capacity. Interestingly, our results indicated that SalB significantly enhanced basal respiration, ATP production, uncoupling capacity and glycolysis capacity as well as promoted the 3T3-L1 cell differentiation. Furthermore, SalB also strikingly increased the morphology and quantity of mitochondria especially after the addition of GW9662 (PPARγ antagonist) in 3T3-L1 adipocytes.

Adipose tissue is considered to be a major endocrine organ affecting the whole body metabolism, in which oxygen consumption (oxidative phosphorylation) and glycolysis are involved. Fortunately, the assessment of real-time changes in cellular metabolism has been greatly facilitated with the introduction of the XF Seahorse extracellular flux analyzer, which measures OCR and ECAR (Perron et al., 2013). Mitochondrial basal OCR reflects both coupled mitochondria respiration and uncoupled consumption of oxygen, oligomycin represents the portion of basal respiration that was being used to drive ATP production, maximal OCR provides an index of energetic reserve capacity and proton leak can be used as a mechanism to regulate the mitochondrial ATP production (Hartman et al., 2014; Pike Winer and Wu, 2014). Additionally, ECAR is largely determined by the release of lactic acid, which is a product of glycolysis. The glycolytic capacity of cells under basal conditions the reserve upon which cells can draw in the face of increased energy demand. Our present study revealed that GW9662 treatment strikingly decreased basal respiration, uncoupling effect and glycolysis capacity in differentiated 3T3-L1 adipocytes, while GW9662 supplemented with SalB significantly increased mitochondrial OCR and glycolysis capacity. In turn, these parameters were also significantly increased by SalB administration when the activity of PPARγ was inhibited. These results indicated that SalB improves mitochondrial function when the activity of PPARγ is inhibited by GW9662 in the 3T3-L1 adipocytes. Previous studies have shown that PPARγ can attenuate mitochondrial oxidative stress by upregulating UCP2 expression (Zhou et al., 2016), and UCP2 directs the metabolic switch toward glycolysis by activating PFKFB2 (Sreedhar et al., 2017). Consistent with these studies, we found SalB significantly upregulated the expression of UCP2 after inhibiting PPARγ activity. These results indicate that SalB improve mitochondrial respiration and glycolytic function through a PPARγ related pathway.

Emerging evidence has demonstrated that PPARγ is a ligand-activated transcription factor that mainly predisposes the adipogenic differentiation molecular network (Wafer et al., 2017). PPARγ agonists trigger mitochondrial biogenesis in white adipocytes from deficient mice, accompanied by remodeling of adipocyte mitochondria in shape, size, and function (Ducluzeau et al., 2011; Rong et al., 2011; Tol et al., 2016). Moreover, PGC-1α binds PPARγ and stimulates the browning of white adipose tissue, mitochondrial biogenesis and respiratory functions that facilitate energy expenditure (Roberts et al., 2014; Wenz et al., 2016). Earlier research has found that SalB improves glycolipid disorders through regulating PPARγ in high-fat induced obese mice (Zhao et al., 2017). Targeting PPARγ with siRNA can inhibit adipogenic differentiation (Li et al., 2014). In the present study, the PGC-1α expression level, mitochondrial content and expression levels of related genes were significantly reduced in adipocytes treated with GW9662. SalB displays an adverse effect on the protection of mitochondrial morphology, content, and upregulating PGC-1α levels. Furthermore, PGC-1α can activate the expressions of PPARγ, PPARα, and NRF1/2 (Wu et al., 2014), which is reported to activate the mitochondrial respiration and protect against oxidative damage (Wan et al., 2012). Therefore, we speculated that SalB could improve mitochondrial biogenesis and promote 3T3-L1 adipocyte differentiation at least in part through the pathway of PGC-1α.

Taken together, our results suggest that treatment with SalB exerts some protection on mitochondrial dysfunction and adipogenesis through a pathway involving PGC-1α. Furthermore, these also raise the possibility that SalB could be useful as an alternative medicine against obesity and its associated diseases.

## Author Contributions

YP and WZ conceived the study and drafted the manuscript. NY and YP carried out the experiments and performed the statistical analysis. TA, JM, YG, and BL contributed reagents, materials, and analysis tools. FM, JL, CW, and DZ analyzed the data. SG and GJ designed the experiments and reviewed drafts of the paper. All authors gave their final approval for publication.

## Conflict of Interest Statement

The authors declare that the research was conducted in the absence of any commercial or financial relationships that could be construed as a potential conflict of interest.

## Supplementary Material

TABLE S1 Oxygen consumption rate (OCR) of different concentration SalB in 3T3-L1 adipocytes.

TABLE S2 Oxygen consumption rate of SalB (50 μM) with or without GW9662 (20 μM) in 3T3-L1 adipocytes.

TABLE S3 Extracellular acidification rates SalB (50 μM) with or without GW9662 (20 μM) in 3T3-L1 adipocytes.
